# Facilitators and barriers to the provision of type 1 diabetes inpatient care: An interpretive phenomenological analysis

**DOI:** 10.1002/nop2.699

**Published:** 2020-11-24

**Authors:** Monica Nikitara, Costas S Constantinou, Eleni Andreou, Evangelos Latzourakis, Marianna Diomidous

**Affiliations:** ^1^ Department of Life & Health Sciences University of Nicosia Nicosia Cyprus; ^2^ Department of Public Health National and Kapodistrian University of Athens Athens Greece

**Keywords:** diabetes, nurses, nursing, type 1 diabetes

## Abstract

**Aim:**

The aim and objective of this study was to understand how non‐specialized nurses understand the possible barriers and facilitators of inpatient care for type 1 diabetes.

**Design:**

An interpretative phenomenology approach was conducted.

**Methods:**

The sample consisted of non‐specialized nurses (*N* = 24) working in medical, surgical and nephrology wards in the state hospitals in Cyprus. The data were collected during 2016‐2018 from one focus group with nurses (*N* = 6) and individual semi‐structured interviews with nurses (*N* = 18) conducted. The Standards for Reporting Qualitative Research checklist used to ensure the quality of the study.

**Results:**

It is evident from the study findings that nurses experience several barriers in diabetes inpatient care reported which are of great concern since this could have adverse effects on patients' outcomes. Only one facilitator has been reported by few nurses.

## 1 INTRODUCTION

Diabetes is an important public health problem, and one of four non‐communicable diseases world leaders has targeted for priority action (World Health Organization (WHO), 2016). Over the last few decades, the number of new incidences of people with diabetes is increasing (Saeedi et al., 2019). According to the International Diabetes Federation, in 2017, almost 425 million adults (20–79 years) had diabetes and the federation also predicted that the number could rise to 629 million by 2045 Key points for IDF Diabetes Atlas, 2017, 2018). This places pressure on the health care of patients with diabetes. Epidemiologic studies suggest that the incidence rate of T1DM has been growing worldwide (International Diabetes Federation, 2013; You & Henneberg, 2016), but the impact of this type is often overlooked as focus has been on the type 2 diabetes epidemic (Tao et al., 2010). However, people with T1DM suffer for a longer period of time and may be admitted to hospitals for diabetes‐related or unrelated conditions which can altered their normal routine (Hillson, 2016).

The literature on outpatient care of patients with diabetes is quite rich, but scientific information on inpatient care is lacking. To fill this gap, this study explored the experiences of nurses in inpatient care. The main aim was to understand and expand knowledge from their perspective on how inpatient diabetes care could be improved.

## 2 BACKGROUND

Various studies confirm that a patient with diabetes mellitus (DM) is more prone to stay longer in the hospital in comparison with a patient without having a history of diabetes, despite the fact that they may be admitted to the hospital with the same diagnosis (Comino et al., 2015; Levy & Dhatariya, 2019; Ostling et al., 2017). Medication errors during hospitalization are a serious problem, with 260,000 people with diabetes in England having experienced an error which could have resulted in serious harm or even death (Health & Social Care Information Centre, 2017). This is a large cost for the governments as diabetes requires a huge budget for prevention as well for treatment, which includes costs for treating diabetes complications in outpatient settings, emergency departments, hospitals or long‐term care facilities. However, the largest percentage of the budget for diabetes is related to inpatient and outpatient care (WHO, 2016).

Despite the large number of people with diabetes who are using the inpatient services, there are still concerns about the quality and safety of the care they receive. This has been documented in the past showing that people with diabetes experience poor inpatient care due to lack of infrastructure (Pledger, 2005). This is also supported by more recent findings which showed that hospitalized people with diabetes did not see a diabetes care team due to understaffing and lack of process (Health & Social Care Information Centre, 2017). Furthermore, recent evidence supports previous literature that nurses might provide poor quality of care and this could be due to several reasons such as lack of knowledge and lack of time (Cardwell et al., 2016). However, that is worrying because, during the last decades, governments have taken many initiatives to support this group of people, especially by giving extra attention to primary care and prevention, whereas they have paid little attention to diabetes inpatient care and to people with type 1 diabetes mellitus (T1DM).

In response to the need for enhanced support of patients with diabetes, multiple changes have occurred in the treatment and care of diabetic patients and in nurses' role which aims to face the increasing rate of diabetes morbidity. Such changes include the establishment of the position of the diabetes specialist nurse (DSN), which allows nurses to prescribe medicines in countries like the UK, to be involved in the various levels of the healthcare system and to not be confined to hospitals (Carey & Courtenay, 2010). This development has been found to help clinical outcomes, to decrease unnecessary referrals to secondary care and to decrease outpatient attendances (Riordan et al., 2017). Nevertheless, it is worth mentioning that, although many countries adjusted the diabetes specialist nurse to their healthcare systems, nurses' roles and work settings differed among countries. For example, in Sweden and the Netherlands, almost half of the diabetes specialist nurses work primary care or in integrated care, while they also have the right to prescribe for people with diabetes. In contrast, most DNSs in Ireland are working in hospitals in the secondary care role and not all of them can prescribe (Riordan et al., 2017).

Therefore, taking into consideration that nurses play a major role in patient care and the current situation of diabetes care, there is a need to explore and understand what facilitators and barriers non‐specialized nurses face when they provide inpatient care for T1DM. Therefore, our key question for research was formulated as follows: How do non‐specialized nurses view the facilitators and barriers to perform their role in diabetes inpatient care?

## 3 RESEARCH METHODOLOGY

### 3.1 Study design

The current study reflects interpretivism epistemology, and it was informed by phenomenological social theories and strictly followed the IPA methodology to better understand how non‐specialized nurses, through their experiences, perceive the facilitators and barriers affecting their role in diabetes care.

### 3.2 Sample population

In line with the theoretical underpinnings of IPA, the participants’ sample for this study was purposive and homogenous. More specifically, 24 nurses who shared the same experiences participated in the current study. Two sources for gathering data were used namely focus groups and interviews. One focus group with six nurses working in a range of specialities and following that, to gain a deeper understanding of their experiences, 18 individual interviews with nurses working in medical, surgical and nephrology wards were conducted. The sample covered the entire area of Cyprus, since participants were recruited from each city in Cyprus. This research followed the general principles of research ethics, consent, anonymity and confidentiality. All the necessary approvals and licences were granted from the responsible bodies.

### 3.3 Methods for gathering data

This study, reflecting interpretivist epistemology and IPA methodology, used two methods for gathering data: focus groups and interviews. Although in IPA studies it is not common to use several types of data collection, triangulation with use of focus groups and interviews add to the richness of the data collected and this could be a strength of this study (Lincoln & Guba, 1985). Therefore, combination of these two qualitative methods was used for confirmation and completeness.

To get access to the nursing sample, it was necessary to have a licence from the Ministry of Health and the directors of the public hospitals to enter the hospitals. After obtaining a licence, the researcher met the persons in charge of the medical, surgical and nephrology wards of the public hospitals and left with them the information leaflet about the study. The researchers decided to approach nurses who work in medical, surgical and nephrology wards since people with diabetes are most often admitted in these wards. The information leaflet provided the researcher's contact details, so anyone who would like to participate in the study could contact the researcher directly to talk through any issues or ask questions about participating. All of the participants were asked for their consent to be in the study. Nurses replied either by posting or faxing the consent form back to the researcher or by responding to the researcher's email address. The intention at the outset of the current study had been to recruit five nurses from each of the wards. For the first stage of the study, the first six respondents were asked to participate in the study which included a focus group to guide the interview questions. In the second stage, eighteen nurses accepted to be interviewed.

The focus group followed a predetermined schedule of topics, which allowed the participants to describe and discuss their experiences of diabetes inpatient care. The participants were asked questions based on the study to gather information to help in making decisions regarding the research. This gave the researchers the opportunity to make the interview guide, to include additional probes for the individual interviews and to enrich the sources of data in depth. Also, the researchers had a chance to became more familiar with how participants experience the topic. The reason for having only one focus group for the nurses was because of the low response rate from them to participate in focus groups and because it was difficult for the participants to agree on a common date and time for the focus group. That was a very time‐consuming process that delayed the progress of the study. After the completion of the focus group, for the second stage of the study, the researcher continues with the semi‐structured interviews with 18 nurses. The duration of the interviews was approximately 45 min to one hour. An interview schedule to help guide the discussions in the interviews was developed for the purposes of this study.

To ensure rigour, the current research is focused on five criteria (credibility, transferability, dependability, confirmability and reflexivity) to ensure quality, since this is a qualitative method and is also based on the participants' experiences. The first four criteria introduced by Guba in 1980s and reflexivity introduced by Berger (2015) which argued that trustworthiness is affected by whether the researcher is would be a member of the group that is being researched and therefore shares the participants’ experience.

### 3.4 Data analysis

**FIGURE 1:**
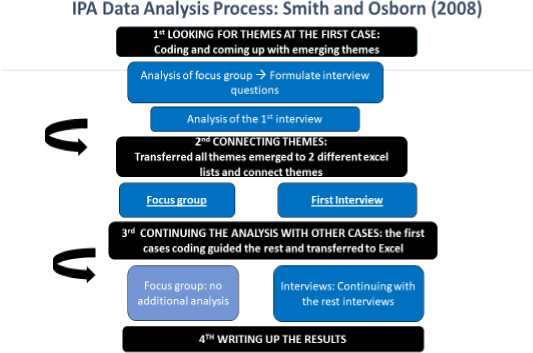
Data Analysis Process

In Smith and Osborn (2008), four stages of analysing data were described which can help to identify shared experiences across a group of participants. Because the current study sought to identify shared experiences of nurses, the following process was taken by two researchers independently to ensure the quality of coding (Figure 1).

#### 3.4.1 Looking for the themes of the first case

At this stage, the researcher spends time analysing the first transcript which was the focus group. The researcher through constant reading of the transcript came to the final themes. These themes helped the researcher to formulate the interview schedule for the semi‐structured interviews with nurses. After completing the semi‐structure interviews with nurses, the researcher started to analyse the interviews. The focus group was used as the first transcript and guided the coding of the interviews. Therefore, the researchers used the noted codes and themes from the focus group to orient the following coding and analysis.

#### 3.4.2 Connecting themes

The researchers brought together all the themes that were previously identified and transferred them into an Excel document prepared specifically for the study. After clustering the themes, the researchers prepared a table with the final themes. Exactly the same procedure was followed as for the first interview with nurses.

#### 3.4.3 Continuing the analysis of other cases

The researchers decided to use the first case to orient the subsequent analysis. Because there was only one focus group with nurses, there was no continuing analysis. The same exact procedure was followed for the interviews with nurses. The themes that emerged from the focus group were used to orient coding and subsequent analysis of interviews. After analysing all nurses' transcripts, the researchers developed a final table with the dominant themes and tried to consolidate them.

#### 3.4.4 Writing up the results

The final step is to translate the analytic themes into a narrative account. Therefore, the writing up stage can be found in the next chapter, which is the analysis of the data and where the themes are written in a narrative way.

### 3.5 Ethical considerations

Research has received a clearance letter from Cyprus National Bioethics Committee. Additionally, licence acquired by the Ministry of Health and the directors of the public hospitals to enter the hospitals and leave with them the Participant Information Sheets (PIS. All the participants were assured that they would remain anonymous, that all of their personal information was confidential and that their participation in the study was voluntary. Nurses were informed that they could withdraw at any time during the project without this affecting their work status. The Standards for Reporting Qualitative Research checklist used to ensure the quality of the study.

## 4 FINDINGS

### 4.1 Nurses' views about facilitators and barriers to diabetes care

Exploring the research question about the facilitators and barriers to diabetes care, the following themes emerged: availability or time, resources, protocols, knowledge, experience, task‐oriented care, integrated care, patients' compliance to self‐care measures and patients’ trust in nurses. Let us now analyse each of the theme separately.

### 4.2 Availability of time

When nurses were asked about factors that help or prevent them from providing adequate care to their patients, most participants indicated that the limited amount of time available was an important barrier. Almost all the participants expressed their dissatisfaction with the heavy workload they have and the lack of time to perform several nursing activities related to diabetes care, particularly concerning diabetes education. Some of them reported that, although they recognized that their patients have multiple needs, including the need to learn about diabetes, there are activities that take priority. Consequently, because of the lack of time and the heavy workload, some jobs are left undone:

I believe that there is insufficient time for the full information/instructions. (2‐F‐45‐Pa‐Me)

It's because of the heavy workload…so I do not have time to catch a person and tell him you have to do these things. You cannot do these things in the medical department. (3‐F‐31‐La‐Me)

Because we have shortage of staff, we have a lot of responsibilities and that [there is] no time to sit down to answer questions. (5‐F‐30‐Li‐Me)

### 4.3 Resources

Nurses working in renal and surgical wards consistently described the difficulty that they face regarding the limited availability of resources. Some of them mentioned the lack of equipment, which prevents them from providing effective diabetes care. They expressed concerns about the resources available, including insulin pens, a blood glucose meter and treatments to take care of wounds. Nurses also recognized the difficulty in providing care or education because of the environmental constraints which leave very little space between other patients and consequently offer no privacy. Interestingly, none of the nurses working in medical wards referred to this theme (lack of resources):

Something I was seeing earlier and made me angry was that they were sticking patients with the needle. In depth. Because the service does not provide us with insulin pens. (10–43‐F‐Am‐Su)

If you come to see how the haemodialysis unit is, the space does not even allow you to undress the patient. If I go into the process of getting their socks off to check their feet, I will expose patient. (14–30‐F‐Ni‐Re)

Lack of consumables is a big problem. There is no blood glucose machine. And we have to borrow it from the other units with difficulties since they need it as well, there are no insulin pens and we have to stick patients with the small orange needles. (15–33‐F‐Ni‐Su)

### 4.4 Protocols

Participants had contradictory views regarding the existence of protocols. Some of the participants coming from different hospitals and ward specialities stated that there are some general guidelines regarding diabetes care and specifically diabetes education. Moreover, a diabetes specialist nurse who is working in the medical ward in a specific hospital reported that all the nurses are following protocols that include detailed information about diabetes care and education. Nevertheless, several participants from other hospitals emphasized that there were no protocols although they need them. Furthermore, the head nurse with the longest experience of the participants said that they lack protocols and even if there were some general procedures, nobody would follow them. There is no control, which leads to dissimilarity in the care nurses provide. Other participants agreed with this view that there are no protocols and that they are not updated about new guidelines. More specifically, 11‐48‐F‐La‐Sup explained the following:

There is no proper protocol to follow in which they will all do the same…to do it and to have someone who controls them and tell them that you did it right or wrong, or we need improvement or not, or we left something out….

Another participant agreed that the information that only a few of the nurses are informed when new guidelines are published, whereas the rest of them are not informed. The following quote from a focus group participant is illuminating:

They do not come to teach us the new guidelines. It is not possible only 5–6 people to go and the rest of the hospital has no idea what is going on. What are the new things that have come up?

### 4.5 Knowledge

Nurses understood their deficit in knowledge was a barrier to them for providing good quality diabetes care and education. Several participants reported to have limited knowledge regarding treatment of diabetes, especially knowledge about insulin and other antidiabetic drugs. When participants were asked if they feel comfortable with their level of knowledge about diabetes medication, most of them reported that they know the basics. For example:

In the theoretical part not so. I know the basics. How do the pills work? No. I know the complications. (7‐35‐F‐Ni‐Me)

Uh… I know in general terms… okay… the basics… more in depth I think it is more medical… and because we receive instructions from the doctors in combination with our knowledge we proceed accordingly. (4‐F‐30‐La‐Me)

Furthermore, some nurses acknowledged that they were not comfortable with their knowledge regarding the pathophysiology and symptoms of diabetes, and diet and education. More specifically, when participants were asked if they felt confident to explain things to diabetic patients, some of them responded with the following:

I know some symptoms but nothing more. I do not know further to explain. Even a doctor may not know how to explain. (1‐F‐24‐Pa‐Me)

For all the symptoms I cannot know… I do not know the pathophysiology of diabetes mellitus very well. (7–35‐F‐Ni‐Me)

### 4.6 Experience

The years of experience nurses had was mainly seen as facilitating them in caring for diabetes patients. Most of the participants expressed their confidence in providing diabetes care and education due to the length of time they had been dealing with diabetic patients. 3‐F‐31‐La‐Me and 6–43‐M‐Li‐Me explained the importance of exposure to incidents on a daily basis:

Yes—but through experience—not from school.—Yes, I have no problem… okay as a medical ward we have many incidents that make you get more experience. (3‐F‐31‐La‐Me)

There is no day in the department that I will not see an incident of either hypoglycaemia or hyperglycaemia or infection on a diabetic foot or an admission with unregulated [glucose]. I see them every day. I can I handle an incident. With my own eye, I recognize. (6–43‐M‐Li‐Me)

### 4.7 Task‐oriented care

An interesting finding is that most of the participants recognized that they were working in a task‐oriented manner to complete jobs such as giving food and medications, making beds and caring for wounds rather than educating diabetic patients or talking to them. This was expressed by nurses working in different hospitals and different ward specialities. Most of them linked this with the limited time available and that they had to complete other important tasks. Other participants referred to the absence of a holistic care approach and as a result they could not meet the individual needs of the patients. Two of the participants indicated that there were nurses merely going through a routine on the ward and doing their job mechanically. Additionally, participants mentioned that nurses in Cyprus were unclear about their duties and needed formal clarification about their roles. The following words show how nurses focus on a task‐oriented role rather than on a more holistic care approach:

You cannot see each patient holistically and take care of the time needed because you do not have the time. (17–35‐F‐Ni‐Re)

No, I will not stop my job to educate a patient… I go there to do the job I have to do and I can do a little teaching. (16–35‐F‐Ni‐Re)

We cannot have personalized care or we do not have the appropriate staff to do that. To work in depth with patients with diabetes for example. (15‐33‐F‐Ni‐Su)

Because we are few, if there are people who are not interested in the job and just come to spend time doing their job mechanically’ (15‐33‐F‐Ni‐Su)

Most people are working mechanically. They finished school, they are leaving everything… and nothing else… (6‐43‐M‐Li‐Me)

### 4.8 Integrated care

One of the important findings is that there is little or no collaboration between people with diabetes expertise and the nurses working in wards. Participants reported that there is no collaboration between the diabetic nurse and the medical ward, which has a negative impact on patients' care and education. 6‐43‐M‐Li‐Me, for example, maintained that:

But we do not have this cooperation between us… so the whole treatment of diabetic patients is lagging behind. That the diabetic clinic, at least in our department, does not have such good cooperation with my department…. They are independent…. (6‐43‐M‐Li‐Me)

Another nurse working in a medical ward said she did not know that there was a diabetic nurse available or that a diabetes clinic existed in the hospital where she worked.

Furthermore, nurses talked about the absence of specific specializations such as psychologists, dieticians and angiologists who have expert knowledge in diabetes, and an inadequate number of diabetic nurses. Also, they referred to the lack of a diabetes team. An interesting plea was made during the nurse's focus group regarding cases of disagreement between physicians and nurses. Specifically, they said that when they disagree with physician's instructions regarding insulin units, they give to the patients what they think is good for the patient and yet they will write in the treatment chart something that is in accordance with the physicians’ instructions. We understand that this is an ethical issue that raised concerns about patient safety; however, we do not want to breach the confidentiality of our participants. Furthermore, this thesis will be sent to the relevant bodies of the country and they will be aware about this issue.

### 4.9 Patients' compliance to self‐care measures

Almost all of the participants referred to the difficulty of providing diabetic care and described some cases where the patients did not comply or resisted accepting any support from the nurses. Some of the participants mentioned that elderly people had low adherence to prescribed self‐care measures because of the inadequate care provided at home by their caregivers. Additionally, they referred to teenagers’ low adherence when they were newly diagnosed because they did not accept their disease and they reported that they had seen patients who were indifferent towards their treatment and care. Two of the nurses working in surgical wards described their experiences with newly diagnosed patients who had a negative reaction, making the nurses' task of care difficult. To illustrate, 12‐33‐F‐La‐Su and 13‐32‐F‐La‐Su unfolded their experiences as follows:

In the beginning he was negative, he was not talking to us, we let him for a while to assimilate himself that diabetes is not something that is bad, it is something you control and can live with it. (12‐33‐F‐La‐Su)

We had a patient who had to go on to Lantus, from pills. He did not accept that. We have many patients who decide on their own that their doses are too many or do not want to take them, sometimes we have such incidents…. (13–32‐F‐La‐Su)

The focus group also supported this observation, saying that most of the patients who were in denial were also unhappy with the nurses’ behaviours. Additionally, nurses working in renal wards said that patients refused to adhere to their treatment and did not listen to the nurses since they believed that they knew better than the nurses about their condition. In particular, two of the nurses working in renal ward said:

They think that the nurses are their soldiers and that we have to do what they are saying…. They are very tiring because they learn some things and they want to do them, while they have to be our soldiers… (18‐35‐F‐Am‐Re)

He may not be in a situation where you can talk to him and give him the right instructions. Especially Cypriot patients, we have too many acting like that. And most of the times these patient who are in denial have also the grievance that we do not have proper behaviour. (Focus group)

#### 4.9.1 Patients' trust in nurses

One view that was frequently expressed by nurses was that patients underappreciate them or did not trust them as much as they trusted physicians. This emerged largely from the focus group discussion, but also during interviews from three nurses working in medical, surgical and renal wards. The focus group described how the Cypriot patients were prejudiced against nurses and underestimated their abilities, believing that a nurse's job was to change patients’ diapers. Two nurses from the focus group explained that:

And unfortunately most of the Cypriots come with prejudice in the hospital. We are very undervalued. Most Cypriots believe that a nurse is the physician's pawn. (Focus group)

Many people say: Okay what are you going to do? You will change the diapers of the patient? What else do you have to do in your work? (Focus group)

This is further supported by a nurse working in a medical ward who stated that she did not educate diabetic patients because patients showed greater trust towards physicians and because they underestimated and doubted nurses’ knowledge and skills. She believed that patients thought that nurses were inferior to doctors. Another nurse, working in a surgical ward reported that patients usually did not trust young nurses. A nurse working in a renal ward stated that diabetic patients did not trust them because they thought that they knew better about their own disease and that nurses working in the renal ward did not know anything about diabetes. The following words reveal nurses' disappointment in how patients’ understand nurses’ skills and capabilities:

Most patients show more trust in doctors than in nurses. They do not give us the necessary attention. They are underestimating us. They think nurses are inferior to doctors, say they are doctors' assistants. (5‐F‐30‐Li‐Me)

Some people, because of age, do not accept that, especially the younger ones, who go to teach them. (13‐32‐F‐La‐Su)

They do not trust 100% what we say. They think they are knowledgeable. They do not let you get into the field of diabetes, they think they are for another specialty, that it's a long way from us. They do not show confidence in me. (14‐30‐F‐Ni‐Re)

### 4.10 Summary

It is obvious from the above analysis that nurses experience multiple barriers when caring for people with diabetes during hospitalization. These barriers have been identified mostly by all the nurse participant's despite the fact that there were differences between their ward specialities. These barriers are of great concern since this could have adverse effects on patients’ outcomes. In following chapter, there will be an extensive discussion about all the results of the current study.

## 5 DISCUSSION

### 5.1 Facilitators and barriers to inpatient diabetes care

Several barriers have been identified by our participants in the provision of diabetes care by nurses in the hospital setting. Nurses’ “lack of knowledge” of diabetes care has been well documented in the literature in previous studies, and our findings add to the evidence. Furthermore, numerous studies have been conducted to identify what factors influence nurses' knowledge acquisition for diabetes care (Alotaibi et al., 2018; Eaton‐Spiva & Day, 2011; Hollis et al., 2014). Despite the plethora of repeated evidence of the nurses' lack of knowledge and the risk for patients’ outcome, nothing was found in the literature about initiatives or developments announced by professional bodies regarding how to tackle this issue. It can be said that the development of the diabetes specialist nurses’ role is something that could amend the situation because of their expertise knowledge, however, their roles and work settings differed among countries, with some of the countries having them available only in the primary settings. Therefore, the care provided to inpatient services by non‐specialized nurses who do not have adequate knowledge is still questionable since there are non‐universal and announced measures about dealing with this issue.

Knowledge and “experience” in diabetes care and its management are crucial for caring and teaching patients with diabetes effectively (Lawler et al., 2019). Our participants thought of their years of experience as a facilitator that allows them to provide efficient diabetes care which is reasonable. Theoretically, nurses with more years of nursing experience are expected to provide higher quality care that is more efficient and effective and thus positively affects patient outcomes, and lowers consumption of resources (Audet et al., 2018). However, as Benner said, that although most nurses will be able to achieve the competent level, that does not mean that all of them will become experts (Benner, 1984). Many authors have differentiated the experience from expertise by explaining that not all experienced nurses are experts (Paans et al., 2017). Therefore, one could assume that the years of experience might bring one to a competent level but it does not necessarily mean that one has achieved a level of expertise because one is repeating the same actions for years.

Furthermore, one could assume that through “continuing education” they could have enriched their knowledge. The Cyprus Nursing and Midwifery Council now requires every nurse to renew her/his practice licence every four years and to show evidence of 32 educational hours or 20 international continuing nursing education credits that have been completed within the last four years. This is still much less than the specified educational hours in other developed countries, which on average are about 24 hr a year. However, the findings from this study suggested that there are limited opportunities for our participants to attend such courses, due to organizational factors or lack of motivation. Therefore, despite the new regulations which are supposed to motivate nurses to continue their education, there are still issues that need to be reviewed and revised in order for both nurses and patients to benefit.

The World Health Organization's 2000 report on health systems described health systems as all the actions that intend to promote health, restore health and maintain population health. One of the most important factors that determine how people view their quality of life is the quality of healthcare (CGI, 2014). At the same time, the main concern of governments around the world is the challenge to find ways to provide health care that is both affordable and high quality (Williams et al., 2019) and healthcare and health systems all over the world are undergoing intensive reforms (Durrani, 2016). Therefore, it was predictable that findings from the current study would have added to these issues, since most of the participants with diabetes highlight the failure of the healthcare system to provide them with adequate care and they blame the structure of the system, even though the Cyprus healthcare system is under transition.

The findings of our study add to the growing body of literature evidencing that one of the most reported barriers nurses encounter in performing their roles is the “lack of time” due to understaffing. Several studies conducted in several countries have proved that the shortage of nurses is associated with higher mortality rates and increases adverse patients' events (Fagerstrom et al., 2018; Griffiths et al., 2019; Son et al., 2019,). The question is that, although understaffing in the nursing profession remains an issue in many countries, the quality of nursing care and the potential for poor nursing care to do patients great harm has been the focus of numerous reports in the past (Care Quality Commission, 2011; Health Service Ombudsman, 2011). At the same time, governments try to face the challenge to reduce costs on health care and at the same time to maintain the quality and safety of healthcare systems (Ball et al., 2014). Subsequently, it is apparent that there is an imbalance between the expected goals of better quality care and the possible strategies developed to achieve them if they include cost reductions, which is certainly related to understaffing of nurses (Everhart et al., 2013).

The lack of time nurses has to provide the necessary care to their patients, and the chronic shortage of nurses could be considered as two sides of the same coin. While there is great concern globally for nurse understaffing, which it is a reason for nurses' lack of time, concurrently, the lack of time could lead nurses to suffer burnout and this in turn will give them the intention to leave their job (Chan et al., 2013; Van der Heijden et al., 2019). Therefore, taking into consideration that nurses' lack of time and understaffing are two sides of the same issue, which is closely related to reduced quality of care provided to patients, with severe implications for them, policymakers of healthcare systems worldwide have to reconsidered their priorities and find solutions to this issue.

Our study is the first to identify that there is a “lack of protocol” for diabetes inpatient care in some of the hospitals in Cyprus. No studies were found in the literature referring to that, which is reasonable, since there have been several, recent developments regarding diabetes protocols. However, there is evidence in the literature that, despite the existence of clinical protocols, nurses fail to follow them (Coats & Marshall, 2013). Consequently, the lack of availability of clinical protocols in conjunction with the nurses’ failure to follow them when they are available could put patients at risk when they have to educate a patient. Additionally, the fact that some hospitals use protocols and some do not show that there is no collective approach to diabetes inpatient care, despite the availability of universal guidelines, protocols and policies for it.

All the above factors could also affect the model where the nurses work. For example, our participants clearly stated that they function in a “task‐oriented” manner. “Functional or task‐oriented” nursing started during WWII when there were not enough Registered Nurses and hospitals had to employ more support staff (Yoder‐Wise, 2018). This model is not liked because it is too regimented and nurses are not able to treat patients as individuals or spend enough time caring for patients’ psychosocial and spiritual needs (Tiedeman & Lookinland, 2004). Opponents to this model support that it is reducing the quality of care, is contributing to fragmentation and is leading to low satisfaction levels for patients (Duffield et al., 2010). The literature supports that wards with poorer skills were more likely to be characterized by a more task‐oriented and a hierarchical approach (Adam & Bond, 2003). This is confirmed in what was said earlier about Benners' theory that not all experienced nurses are experts, but they will be competent nurses who are task‐oriented and deliberately structure their work in terms of plans for goal achievement. Given that diabetes affects aspects of patients' lives, it is preferable that inter‐professional providers take a holistic approach which uses their specific knowledge and skills to provide quality care. One could assume that task‐oriented care does not encompass the necessary features to deal with the global burden of chronic diseases and specifically diabetes as it does not focus on individuals but on tasks to be completed.

What was obvious from the findings of the current study is that there is a lack of “integrated care” in diabetes care, with participants referring to the lack of collaboration with diabetes specialists or even the absence of certain specialities. It is evident from the literature that health practitioners need to have access to the necessary skills and technology to adequately care for their patients and help them to achieve their therapeutic goals (Bolsin, 2014). Because care is not integrated, health practitioners must refer to others to achieve their therapeutic or treatment goal, which is called “therapeutic partition” (Nancarrrow, 2015). Therapeutic partitions cause patients to engage in more clinical transactions which can add more expense and time and can negatively affect care they receive compared with an intervention provided by a co‐located team (Gucciardi et al., 2016).

To tackle this issue, there is a trend in other countries to integrate the care of diabetes because of the complex nature of the disease. Also, the need for an inter‐professional team approach is known to be essential for diabetes management (Gucciardi et al., 2016). Even though, the traditional model for diabetes care is about primary and secondary care, integrated diabetes care aimed to put individuals at the centre of the care and omits any obstacles between organizations and specialties. This approach will bring better outcomes for the patients but will also achieve a better value for the system (Diabetes UK, 2014). Clinical studies supported that the introduction of diabetes specialists' teams in hospitals will have better patient outcomes, a reduction in prescribing errors, a reduction in patients' lengths of stay at hospitals, an increase in the day case rates and consequently hospitals will have a reduction in the admission rates (NHS Diabetes, 2013).

Integrated care, unlike the old model which was more like a supply‐driven provision of care, centralizes the patients in the service delivery and views them as the organizing principle (González‐Ortiz et al., 2018). Establishing “vertical integration” in diabetes care will result in the reduction of duplication or gaps in the services between primary, intermediate and secondary levels of care. In integrated diabetes care, the needs of the patients and their families should be the primary focus of professionals and this can be achieved by incorporating a healthcare system which will provide coordinated services to this group of people (NHS Diabetes, 2013). While it is obvious from the current findings that there is a fragmented continuity of diabetes care, evidence showed that there is a need for integrated diabetes care which will offer an organized and holistic provision of care with emphasis on the continuation and coordination of the provided services. Therefore, it is something that needs further investigation and maybe a restructure in the delivery of diabetes care. Our study provides evidences about the importance of additional expertise of nurses and other healthcare professionals in different departments and diverse fields of medical and nursing care clinics which would have a significant impact on the provision of integrated care. Additionally, patients’ “lack of trust” in nurses was one of the most reported comments by the nurse participants of this study. According to many nursing councils, such as the UK Nursing and Midwifery Council, trustworthy in nurses is a formal expression in their code of Professional Conduct (Nursing & Midwifery Council, 2004). Studies that estimate patients' level of trust in nurses in other fields and not specifically in diabetes care indicate that nurses are highly trusted by patients (Milton, 2018). Our findings are not consistent with the literature. For instance, the participants with diabetes in the current study showed they value physicians' roles rather than nurses. In other studies, nurses rated “trusting relationship” items significantly higher than patients did (Tuckett et al., 2009). No studies were found referring to diabetic patients' trust in nurses except for one that related trust in nurses to positive outcomes for people with diabetes, such as better control of glucose levels and better quality of life (Lee & Lin, 2011). This inconsistency between our findings and the wider literature might be due to the fact that medical profession has a privileged position in the Cyprus society while nurses are viewed as the helpers of the doctors with much less knowledge. For patients with chronic illness such as diabetes, trust is an important factor which determines their disease experience and as can be seen from the above, trust is a vital value in nurse–patient relationships. To meet the needs of the patients, nurses should recognize the importance of developing trustful relationships with their patients, as this will be beneficial for both parties. Trusting relationships have been related to patients’ better adherence to treatment and care, and this helps nurses to feel more satisfied with their job and makes them more willing to contribute to their patients' recovery (Dinç & Gastmans, 2013). Therefore, if we assume that the participants' perceptions about the lack of trust are valid, with all the previously stated reasons to hinder this, then the negative outcomes affect both patients and nurses' job satisfaction. Furthermore, there is no other evidence in the literature from other countries that shows that people with diabetes value the roles of doctors more and underestimate the role of nurses. This confirms Loizou et al.'s (2009) findings that people with diabetes seemed to rely to a great extent on their physician not only for their medications but also for psychological support. This is an interesting finding, although general trust in physicians plays a significant role in patient care. Evidence in the global literature has shown that public and patient trust towards their medical profession has seemingly reduced (Huang et al., 2018). Through the last years, the relationship between doctors and patients has undergone a transition. Previously, patients would seek doctors' decisions for their disease and the patients would have to adhere to these decisions. This was a paternalistic model which allowed doctors to use their knowledge and skills and to decide for their patient to reinstate health. These paternalistic interactions have been replaced by a model that engages patients more in their treatment (Armstrong, 2014). Therefore, it is of great interest that despite these changes elsewhere, in the Cypriot context, the medical profession still plays a dominant role and is considered a superior profession compared with other health professionals. This is may be due to the fact that a paternalistic approach still dominates, and doctors have a high status in some societies.

### 5.2 Limitations

This study used a sample from one country which uses a specific healthcare system. One of the drawbacks of this is the possibility of specific bias which reduces the generalizability of the findings.

### 5.3 Conclusion

The literature clearly shows that, on average, a patient with diabetes spends longer time in hospital than a patient without diabetes—despite being admitted for the same procedure or condition. Therefore, it is important to ensure that people with diabetes constantly accept proper and timely ward‐based and specialist care, but this is still under‐researched. Numerous barriers have been identified that inhibit the nurses' roles in diabetes inpatient care and only one facilitator has been reported. Being able to eliminate these barriers in a realistic way could enhance nurses' roles and consequently will affect the quality of care provided to people with diabetes. These effects of improved care could be seen at the national and even international levels, since this could reduce the number of readmissions of patients to hospital, could reduce patients' lengths of stay, could contribute to the reduction of diabetes complications and most importantly could reduce the cost of diabetes care.

### 5.4 Relevance to clinical practice

Having identified that people with diabetes often experience poor inpatient care which leads to extended hospital stays and other adverse effects for their health, the aims of our study have been defined. This helped us to develop an understanding about the facilitators and barriers that affect nurses to clinical practice specifically in relation to T1DM care.

## CONFLICT OF INTEREST

No conflict of interest to declare.

## AUTHOR CONTRIBUTIONS

MN: Substantial contributions to conception and design, acquisition of data, analysis and interpretation of data; drafting the manuscript; and revising it critically for important intellectual content. Agreed to be accountable for all aspects of the work in ensuring that questions related to the accuracy or integrity of any part of the work are appropriately investigated and resolved. CSC: Have made substantial contributions to conception and design. Been involved in drafting the manuscript or revising it critically for important intellectual content. Given final approval of the version to be published. Agreed to be accountable for all aspects of the work in ensuring that questions related to the accuracy or integrity of any part of the work are appropriately investigated and resolved. EA: Academic guidance and critically revise earlier draft of the paper. Given final approval of the version to be published. Agreed to be accountable for all aspects of the work in ensuring that questions related to the accuracy or integrity of any part of the work are appropriately investigated and resolved. EL: Substantial contributions to analysis and interpretation of data. Been involved in drafting the manuscript. MD: Academic guidance and critically revise earlier draft of the paper. Given final approval of the version to be published. Agreed to be accountable for all aspects of the work in ensuring that questions related to the accuracy or integrity of any part of the work are appropriately investigated and resolved.

## Data Availability

Research data are not shared. Only the researcher could access to the stored data.
